# Effects of a Skin Neuropeptide (Substance P) on Cutaneous Microflora

**DOI:** 10.1371/journal.pone.0078773

**Published:** 2013-11-08

**Authors:** Lily Mijouin, Mélanie Hillion, Yasmina Ramdani, Thomas Jaouen, Cécile Duclairoir-Poc, Marie-Laure Follet-Gueye, Elian Lati, Florent Yvergnaux, Azzedine Driouich, Luc Lefeuvre, Christine Farmer, Laurent Misery, Marc G. J. Feuilloley

**Affiliations:** 1 Laboratory of Microbiology Signals and Microenvironnement LMSM, EA 4312, Normandie Université, Université Rouen, Evreux, France; 2 GlycoMev EA 4358, Normandie Université, Université Rouen, Mont-Saint-Aignan, France; 3 Bio-EC Laboratory, Longjumeau, France; 4 BioEurope Research, Anet, France; 5 Dermatologic Laboratories Uriage, Courbevoie, France; 6 EA 4685 University of Western Britanny, Brest, France; 7 Department of Dermatology, University Hospital of Brest, Brest, France; ENEA, Italy

## Abstract

**Background:**

Skin is the largest human neuroendocrine organ and hosts the second most numerous microbial population but the interaction of skin neuropeptides with the microflora has never been investigated. We studied the effect of Substance P (SP), a peptide released by nerve endings in the skin on bacterial virulence.

**Methodology/Principal Findings:**

*Bacillus cereus*, a member of the skin transient microflora, was used as a model. Exposure to SP strongly stimulated the cytotoxicity of *B. cereus* (+553±3% with SP 10^−6^ M) and this effect was rapid (<5 min). Infection of keratinocytes with SP treated *B. cereus* led to a rise in caspase1 and morphological alterations of the actin cytoskeleton. Secretome analysis revealed that SP stimulated the release of collagenase and superoxide dismutase. Moreover, we also noted a shift in the surface polarity of the bacteria linked to a peel-off of the S-layer and the release of S-layer proteins. Meanwhile, the biofilm formation activity of *B. cereus* was increased. The Thermo unstable ribosomal Elongation factor (Ef-Tu) was identified as the SP binding site in *B. cereus*. Other Gram positive skin bacteria, namely *Staphylococcus aureus* and *Staphylococcus epidermidis* also reacted to SP by an increase of virulence. Thermal water from Uriage-les-Bains and an artificial polysaccharide (Teflose®) were capable to antagonize the effect of SP on bacterial virulence.

**Conclusions/Significance:**

SP is released in sweat during stress and is known to be involved in the pathogenesis of numerous skin diseases through neurogenic inflammation. Our study suggests that a direct effect of SP on the skin microbiote should be another mechanism.

## Introduction

Skin is the largest neuroendocrine organ of the human body [1] and it hosts the second most numerous microbial population [2]. There is increasingly strong evidence that bacterial virulence is partly regulated by host hormones and neurotransmitters [3], [4]. By sweat or direct contact with the dermis or the epidermis [5], skin micro-organisms are in contact with host communication factors. It is therefore paradoxical to note that, until now, the interaction of skin neuropeptides with the bacterial microflora was not taken into consideration.

Substance P (SP), the main neuropeptide identified in skin nerve endings, is essentially located in primary afferent C-fibers and is released in the skin [6]. This undecapeptide of the tachykinin family has multiple bioactivities other than neurotransmission [7], such as capillary vasodilatation, fibroblast and keratinocyte proliferation or mast cell degranulation [1]. It is considered a major mediator of neurogenic inflammation [8] and itch [9]. Cutaneous neuropeptides, and particularly SP, contribute to the pathogenesis of numerous skin diseases, like psoriasis [10], atopic dermatitis [11], [12], immediate and delayed hypersensitivity [13], acne [14] or rosacea [15]. These diseases have multifactorial origins and we suggest that SP could also act through interaction with the skin microflora. Indeed, SP has both direct and indirect antimicrobial activities by acting as a weak cationic antimicrobial peptide [16] and by stimulating the release of cathelicidins and defensins [17]. Different skin neuropeptides have antibacterial activities [3] but, as SP, these activities are generally observed at high non-physiologic concentrations (>10^−4^ M). At low doses, peptides, including anti-microbial ones, can affect the bacterial physiology independently of any modification in their growth rate [18]. For instance, at sub-micromolar concentrations some neuropeptides, such as dynorphin [19] or natriuretic peptides [20], [21] have been shown to stimulate bacterial virulence.

In the present study, we investigated for the first time the action of SP on skin bacterial virulence using a human skin strain of *Bacillus cereus*. The mechanism of SP action on *Bacillus cereus* and its binding site were identified. We revealed that SP is acting on other Gram positive bacteria, namely *Staphylococcus aureus* and *Staphylococcus epidermidis,* suggesting that SP should act as a regulator of bacterial virulence in some of the principal skin associated bacteria. We also observed that this effect of SP on bacterial virulence can be antagonized by thermal water and an artificial polysaccharide.

## Materials and Methods

### Bacterial Strains, Growth Media and Culture Conditions

*Bacillus cereus* (MFP01), *Staphylococcus aureus* (MFP03) and *Staphylococcus epidermidis* (MFP04) originate from our library and were isolated previously from the skin of human donors in the framework of an industrial collaborative program. These bacteria, collected under control of the CRO Bio-EC (Longjumeau, France) in agreement with French and EU Ethic guidelines (ARS Biomedical Research Agreement N°2012-12-010, Bioethic Agreement DC-2008-542), have been identified using API® strips, 16S ribosomal RNA gene sequencing and whole proteome analysis by MALDI mass spectrometry and Biotyper analysis (Bruker Daltonics). For confocal microscopy, these bacteria were transformed by insertion of the pTeTON-GFP plasmid [22]. Bacteria were grown at 37°C in Luria-Bertani (LB). For pre-treatment, they were diluted at a ratio of 1∶40 in fresh broth and the peptides were added at the beginning of the log growth phase. Before use bacteria were rinsed to remove traces of the tested molecule. Substance P (SP) and the reversed sequence peptide (SPrev) were obtained from Polypeptide, Strasbourg, France. In all the studies, controls were carried out using SPrev.

### Infection Studies

The interaction of bacteria with keratinocytes was studied using HaCaT cells (Cell Line Services, Eppelheim, Germany). Cells grown at 37°C in 5% CO_2_ atmosphere in Dulbecco’s modified Eagle’s medium (DMEM, Lonza) were starved of antibiotics 24 h before infection assays. Cells were used between passages 41 and 65. The cytotoxic potential of bacteria was determined by measurement of lactate dehydrogenase (LDH) using the Cytotox 96 assay (Promega, Charbonnieres, France). The bacterial activation of caspase-1 was measured using a commercial kit (BioVision RP, Milpitas, California). For cytological studies *B. cereus* was treated with SPrev or SP and monolayers of HaCat cells were exposed to the bacteria at a multiplicity of infection (MOI) of 10∶1. After incubation, keratinocytes were washed to remove unattached bacteria. Cells were then fixed for 10 min in 4% paraformaldehyde in water and permeabilized for 4 min in 0.2% Triton-X 100 in phosphate buffered saline (PBS). Actin was stained with anti-actin rabbit monoclonal antibody (Millipore, Molsheim, France) and revealed using goat anti-rabbit Alexa 633-labelled antibody (Molecular Probes, Saint-Aubin, France). Preparations were observed using a LSM 710 confocal laser-scanning microscope (Zeiss).

### Identification of SP Stimulated *B. Cereus* Virulence Factors

For cereulide assay, *B. cereus* treated with SPrev or SP (10^−6^ M) were extracted with pentane. The solvent was evaporated and the residue was analyzed by HPLC-MS as described by Häggblom *et al*. [23]. Since cereulide is not commercially available, the calibration was made using valinomycin (Sigma, St Quentin-Fallavier, France). For the study of *B cereus* exoproteins, supernatants from bacterial cultures were obtained by centrifugation at 7000× *g* for 10 min. After filtration on 0.22 µm disposable filter units, proteins were precipitated overnight on ice by addition of 10% trichloroacetic acid (TCA, v/v). Proteins were harvested at 13000× *g* for 20 min at 4°C, washed three times in cold acetone and dried for 1 h at room temperature. Extracted proteins were then dissolved in rehydratation buffer [24] in a final volume of 350 µL. The protein concentration was determined on an aliquot by Bradford assay. Proteins were separated on 12% w/v polyacrylamide 1D gel SDS-PAGE and on 2D gels using pH 4 to 7 non-linear IEF strips and 12% w/v polyacrylamide gel [24]. The second dimension separation was run at 50 mA/gel for 4 h. Gel images were captured using a GS-800 densitometer (Bio-Rad) and analyzed using the Bio-rad PDQuest 2D analysis software. Bands or spots of interest were dissected, submitted to in-gel trypsin digestion [24], and analysed by MALDI-TOF using an AutoFlex III mass spectrometer (Bruker Daltonics) and a FlexControl software Version 3.3. The spectrometer was used in a positive/reflector mode using peptide calibration standards (Bruker Daltonics) as references. Samples were spotted onto MTP 384 ground steel targets using freshly prepared matrix solution composed of 2,5-dihydroxybenzoic acid (20 mg.mL^−1^ in trifluoroacetic acid and acetonitrile). Each spectrum was established over an average of 500–1000 laser shots and 2,5-dihydroxybenzoic acid as matrix. The MS peak list was submitted for fingerprinting using Biotools (Version 3.2). The NCBI data base was searched using MASCOT (http://www.matrixscience.com/cgi/nph-mascot.exe).

### Transmission Electronic Microscopy Observations

Control and SP-treated *B. cereus* were studied by Transmission Electronic Microscopy (TEM) using a Tecnai 12, FEI Company microscope. Bacteria fixed in 2% glutaraldehyde and postfixed with 1% osmium tetroxide were embedded in 2% agarose low melting point. After solidification, samples were cut into 2 mm^3^ slices, dehydrated, embedded in Spurr and polymerized for 24 h at 60°C. Ultrathin sections were obtained using a Leica EM UC6 ultramicrotome. The sections were contrasted with 0.5% uranyl acetate and lead citrate (10 min). The microscope was set at 60 kV. Images were processed by the PRIMACEN Platform (http://primacen.crihan.fr).

### *B. Cereus* Surface Hydrophobicity and Biofilm Formation Studies

The polarity of control and SP treated *B. cereus* was studied using the MATS technique [25] and two solvent couples: chloroform/hexadecane and ethyl acetate/n-decane. The effect of SP on *B. cereus* biofilms was investigated using a LSM 710 CLSM (Zeiss). GFP-transformed bacteria grown for 13 h in the presence of SP or SPrev were poured in dishes containing sterile glass slides. After 2 h a slide was heat fixed to observe the initial adhesion. The other slides were covered by LB medium containing SP or SPrev and incubated for 5 or 24 h without agitation. In order to control their structure, the biofilms were scanned every micrometer in depth at 3 random positions. Three-dimensional (3D) images and ortho cuts (3D/z) were reconstructed using Zen® 2009 software (Zeiss). The biofilm thickness was quantified using the same software.

### Identification of the *B. Cereus* SP Binding Site

For identification of the *B. cereus* SP binding site, proteins from mid-log growth phase bacteria were extracted as previously described [27]. Briefly, bacteria were harvested by centrifugation and washed with ice-cold PBS. The pellet was resuspended in non-denaturing lysis buffer (Tris 50 mM pH 8, EDTA 40 mM, NaCl 137 mM, glycerol 10%, Triton X-100 1%, Phenylmethylsulfonylfluoride 1 mM) supplemented with complete protease inhibitor cocktail (Complete Protease Inhibitor cocktail tabs, Boehringer). After sonication, cell fragments were removed by centrifugation and the supernatant was ultracentrifugated at 148,000× *g*, 50 min, 4°C. The pellet was then resuspended in a second ice cold lysis buffer (Tris 50 mM pH8, MgCl_2_ 10 mM, Triton X-100 2%, Phenylmethylsulfonylfluoride 1 mM, Protease Inhibitor cocktail). The SP binding site was investigated by an immunoprecipitation technique adapted from Mijouin *et al*. [26]. SP antibody-associated beads were made by incubating G protein-coupled agarose beads (Millipore) and SP monoclonal antibody (Abcam, Paris, France) overnight. Meanwhile, 1.5 mg of *B. cereus* membrane protein extract was incubated with SP (10^−6^M) for 1 h at 20°C. To reduce the non-specific binding, 50 µg of rabbit polyclonal serum was added. Unlabelled agarose beads were added to fix non specific complexes. The sample was incubated 30 min at 4°C and the beads were removed by centrifugation (14000× *g*, 10 min). The supernatant, containing the SP bond ligand, was then incubated with the SP antibody-associated beads for 1 h at room temperature under slow orbital agitation. Beads were then washed by successive low speed centrifugation cycles in non-denaturing lysis buffer. The ligand was separated from the beads and denaturated by adding loading buffer (Tris 200 mM pH 6.8, glycerol 45%, SDS 6%, β-mercaptoethanol 6%, bromophenol blue 0.03%) and boiling for 5 min. Before loading on 12% w/v polyacrylamide gel SDS-PAGE, the beads were eliminated by centrifugation. Proteins were visualized by colloidal coomassie brilliant blue G250 staining (Sigma). Three independent experiments were conducted. The electrophoretic band of interest was dissected, submitted to in-gel digestion and analyzed by MALDI-TOF as previously described.

### Inhibition of the SP Virulence Stimulating Activity

Bacteria were diluted to a ratio of 1∶40 in fresh broth and exposed at the beginning of the log growth phase to SP or SPrev (10^−6^M) and Uriage Thermal Water (UTW) 30% v/v or Teflose® (TF) 0.1% w/v or SRrev (10^−6^M). After 1 h, the micro-organisms were harvested by centrifugation, washed and the cytotoxic potential of the bacteria was determined at an MOI of 10∶1 by measurement of LDH release. Controls showed that at the concentrations of TF and UTW used had no effect on the growth kinetics of the bacteria or on the survival of keratinocytes.

### Statistical Analysis

Cytotoxicity (LDH), caspase 1 and MATS experiments were conducted independently at least three times at different days. The results are expressed as Means ± SEM and statistical differences were determined using the Student’s *t-*test. Significant differences were noted as ★, ★★ and ★★★ for *p*-values *<0.05*, *<0.01* and *<0.001*, respectively. For each biofilm, the thickness was calculated from a minimum of 20 measures in different fields using the Zen® 2009 software (Zeiss). The mean biofilm thickness was quantified over three different experiments and the Student *t*-test was used to compare the means. SDS-PAGE electrophoresis, bidimensional electrophoresis, MALDI-TOF and immunoprecipitation figures are representative of three independent experiments.

## Results

### Substance P is Stimulating the Virulence of *Bacillus Cereus*

**Figure 1:**
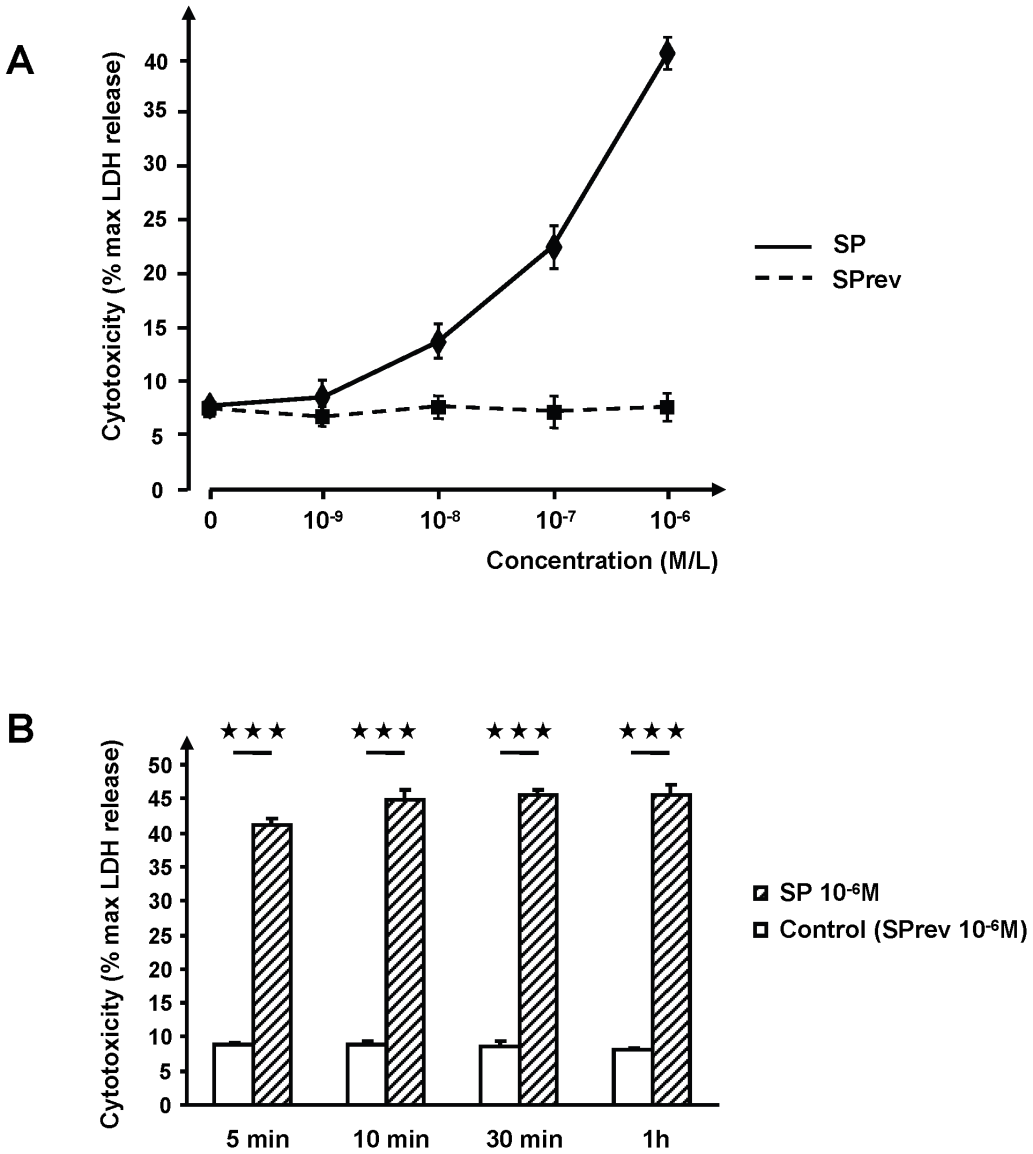
Effect of Substance P on the cytotoxic activity of *Bacillus cereus*. (**A**) Dose response curve of the effect of Substance P (SP) and of the reversed sequence peptide (SPrev). (**B**) Time course response of the bacteria to SP and SPrev (10^−6^ M). (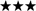 = p<0.001).

The study was realized on *Bacillus cereus* MFP01, a strain identified on the skin of normal human volunteers. Preliminary controls showed that exposure of *B. cereus* to SP (10^−6^ M) during the whole growth phase or at the beginning of the stationary phase, did not modify the growth kinetics of the bacterium. SP was also without effect of the swimming and swarming activities. The peptide was not tested at higher concentrations as it is known to have possible antibacterial activities [16]. When *B. cereus* was grown in the presence of SP, the bacterium showed a strong increase in its cytotoxic potential on keratinocytes, as revealed by the LDH release assay (Fig. 1A). The threshold of SP activity was between 10^−9^ and 10^−8^ M. At a concentration of 10^−6^M, the cytotoxic activity of *B. cereus* reached 553±3% (*P<0.001*) of the control. Since keratinocytes might be sensitive to SP [28], bacteria were carefully rinsed to remove any trace of free SP prior cell infection. In addition, control experiments realised by direct treatment of keratinocytes with SP (10^−6^ M) showed that, in our experimental conditions, SP had no direct lethal effect on keratinocytes and did not modify the cytotoxic effect of SP treated bacteria. The specificity of the effect of SP on *B. cereus* was also controlled by using the SP reversed sequence peptide (SPrev). Even at the highest concentration used (10^−6^ M), SPrev was without any effect on *B. cereus* cytotoxicity. Then SPrev was used as a control for SP during the whole study. The response of *B cereus* to SP was particularly rapid. A 5 min treatment of the bacterium during the mid log growth phase was sufficient to reach 90.5% of the maximal response (Fig. 1B). There was no difference of cytotoxic activity between bacteria exposed to SP for 10 min and 1 h. The cytotoxic activity of the bacteria was not modified after 1 h of treatment.

**Figure 2:**
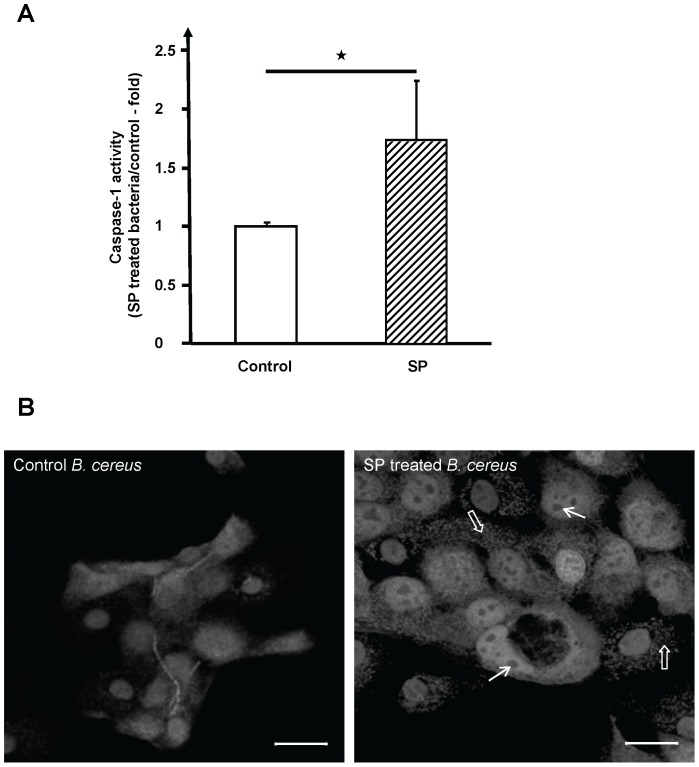
Effect of Substance P treated *Bacillus cereus* on caspase 1 induction and on the morphology of HaCaT cells. (**A**) Cells exposed to SP treated bacteria showed an increase of caspase 1 production (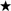 = *p<0.05*). (**B**) After a 30 min infection with control bacteria the morphology of HaCaT cells remained unchanged but when the cells were exposed to SP treated *B. cereus* we observed a complete disorganization of the actin cytoskeleton (□) and the formation of multiple cytoplasmic vacuoles (γ). Bar = 20 µm.

In order to characterize more precisely the effect of SP on *B. cereus* virulence we measured the production of caspase-1 by keratinocytes after infection with bacteria pretreated for 1 h with SPrev (control) or SP 10^−6^ M. As shown in Fig. 2A, after 30 min of infection, the production of caspase-1 by keratinocytes exposed to SP treated *Bacilli* rose significantly (+174±51%, *P<0.05*). Caspase-1 is an enzyme involved in pyroptosis and cell necrosis which is activated during the inflammasome induction [29]. An assay of other caspases and/or cytokines should be necessary to determine the precise inflammatory pathway induced by *B. cereus*. As this process is generally associated with alterations of the cellular morphology, we observed HaCaT keratinocytes exposed to SPrev and SP treated *B. cereus*. After a 30 min infection with control bacteria the histological structure of keratinocytes remained unchanged, whereas the structure of HaCaT cells exposed to SP-treated *B. cereus* was dramatically altered (Fig. 2B). In particular, we observed a complete disorganization of the actin cytoskeleton and the formation of intra-cytoplasmic vesicles. After 1 h infection with SP treated bacteria the HaCaT cell monolayer was completely disorganized. It is interesting to note that keratinocytes exposed to control bacteria for a longer period (5 h) finally presented the same aspect, suggesting that SP not only increased but also accelerated the production of virulence factors.

### Substance P Induces the Release of Superoxide Dismutase and Collagenase by *Bacillus Cereus* and a peel-off of its S-layer

**Figure 3:**
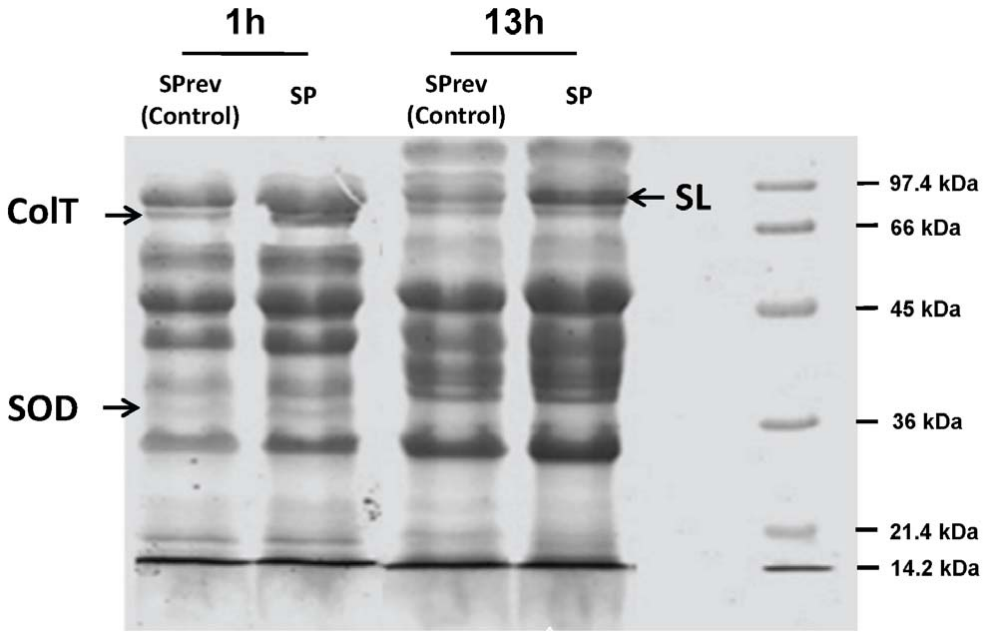
SDS page analysis of secreted Bacillus cereus proteins after 1 or 13(SP) or Substance P reverse (SPrev) (10^−6^ M). Bacteria exposed for 1 h to SP showed an increase in the production of the collagenase (ColT) and of superoxide dismutase (SOD). Bacteria treated for 13 h with SP presented in contrast an increase in the S-layer crystal protein (SL). Results are representative of three independent experiments.

**Figure 4:**
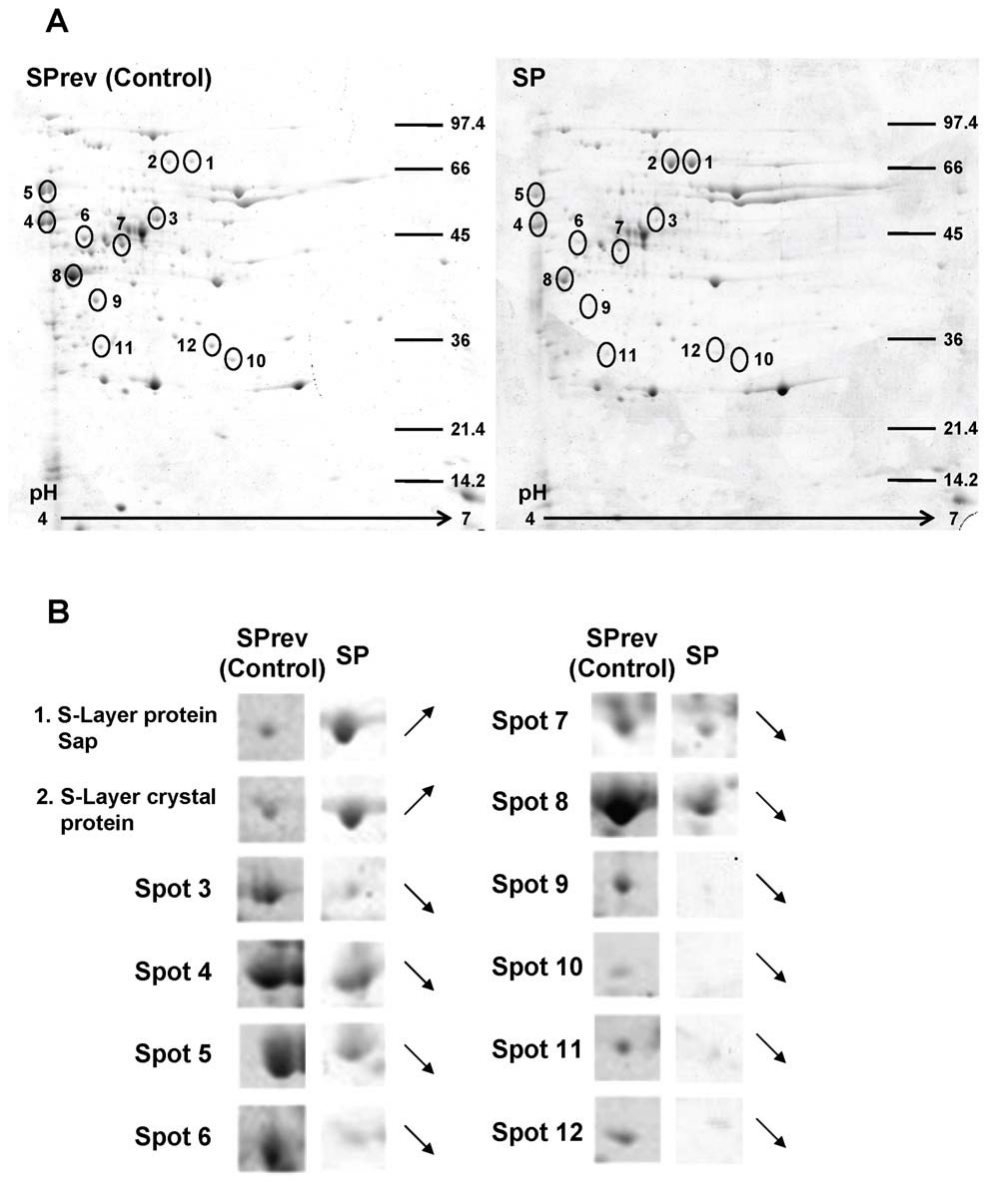
Bidimensional electrophoresis analysis of secreted proteins by *Bacillus cereus* after a 13 h exposure to Substance P reverse (Control) or Substance P (SP) (10^−6^ M). (**A**) Twelve spots were modified after exposure of the bacteria to SP. (**B**) Two proteins (1 and 2) were over produced whereas the expression of 10 others was down regulated. Identified proteins are presented in Table 1. Results are representative of three independent experiments.

The effect of SP appeared mediated through the production of diffusible virulence factors since bacterial cultures and filtered growth medium were equally cytotoxic. As the effect of SP was rapid, we first investigated a possible increase in the production of cereulide, the emetic toxin of *B. cereus* known for its very short kinetic of action [30]. HPLC-MS analysis showed that *B. cereus* MFP01 actually produced cereulide (106 ng/g wet weight of bacteria) but this production was unchanged after SP treatment. Then, exoproteins produced by *B. cereus* were studied. Image analysis of 1-D gels showed variations in 3 major bands between the control and SP treated bacteria, two in the mid-log phase culture secretome and a different one in the late stationary phase (Fig. 3). The first protein over-expressed by 1 h SP-treated *B. cereus* was identified by MALDI-TOF as a 24 kDa manganese dependent superoxide dismutase (SOD) (NCBI number ZP_00239265). The second protein was as the 66 kDa collagenase ColT (NCBI number ZP_04321651). These proteins were not over-expressed by late stationary phase bacteria but we noted an increase of a 87 kDa band corresponding to the S-layer crystal protein (NCBI number YP_002337034.1). These results were confirmed by 2-D electrophoresis. However, *Bacillus cereus* is known to express different types of related S-layer proteins [31] and the PDQuest 2D analysis revealed that in fact two closely related 87 kDa proteins, the S-layer crystal protein (YP_002337034.1) but also the S-Layer protein Sap (HM626283.1), were over produced by *B. cereus* after a 13 h treatment with SP (Fig. 4). Conversely 10 proteins, essentially energetic and intermediate metabolism enzymes, were down regulated by SP (Table 1).

**Table 1:** MALDI-TOF/TOF identification of the proteins in Figure 4 which expression was modified by Substance P in *Bacillus cereus*.

Spot number	NCBI accession number	Genename	Putative function	Protein domain(s)	Mascot score	Number matched peptides	Coverage (%)	Mass (Da)& pI
**1**	HM626283.1	*sap*	Sap (S-layer protein)	Conserved S-layer Homology Domain,bacterial Ig-like domain	352	56	62	87286/5.63
**2**	YP_002337034.1		Crystal protein (S-layer protein)	Conserved S-layer homology domain,bacterial Ig-like domain	246	47	59	87543/5.71
**3**	AE017194	*mmgE*	mmgE protein		239	35	62	43358/5.34
**4**	NP_981531.1	*eno*	Phosphopyruvate hydratase	Enolase	250	29	74	46371/4.68
**5**	ZP_03108403	*groL*	Chaperonin GroL	GroEL-like chaperonin, polypeptide binding site	237	44	72	57384/4.79
**6**	YP_002341293		Sulfatase	Alkaline phosphatase superfamily	185	33	47	73343/6.19
**7**	ZP_04302850.1		Alanine dehydrogenase	Rossman-fold NAD(P) binding proteins	236	33	89	40084/5.22
**8**	ZP_00240745		Bacillolysin	Zn metalloprotease, Funga lysine, M36Superfamily with Zn binding site	88	17	38	54586/5.30
**9**	ZP_04188209.1		Malate dehydrogenase		144	23	68	33535/5.05
**10**	YP_002532873	*hbd*	3-hydroxybutyryl-CoA dehydrogenase	Rossman-fold NAD(P) binding proteins	126	19	59	31269/5.45
**11**	YP_002443562		Pyridoxal biosynthesis lyase PdxS		145	23	72	31855/5.18
**12**	YP_002528490.1	*spH*	Sphingomyelinase c	Metal binding site, phosphate binding site,catalytic site	201	30	69	37462/5.99

**Figure 5:**
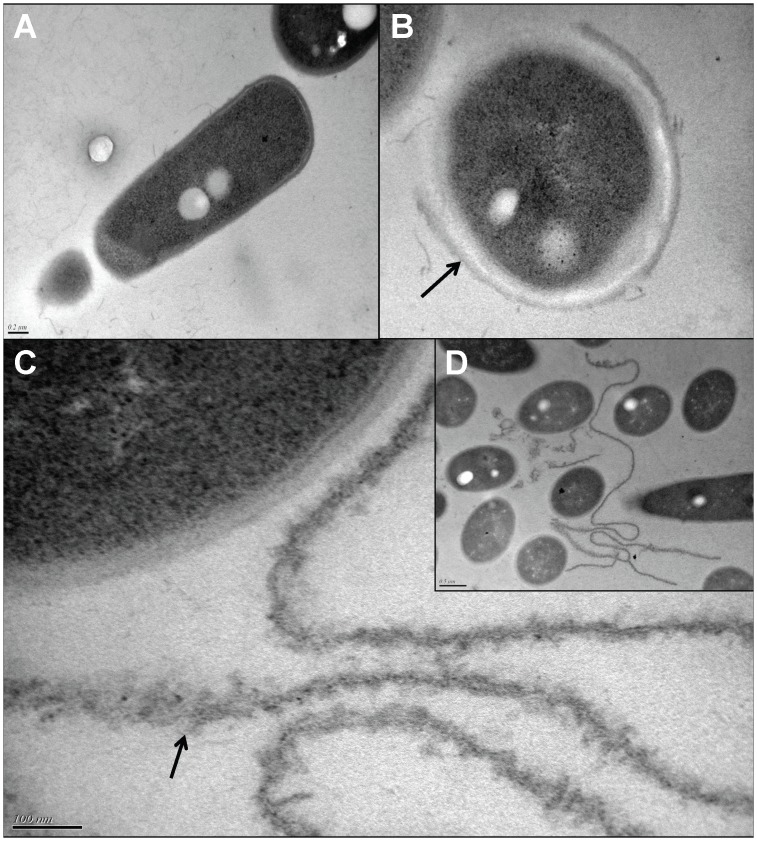
TEM observation of control (SPrev) and Substance P (SP) treated *Bacillus cereus*. Whereas control bacteria presented a continuous intact surface (**A**), bacteria exposed to SP showed a detachment of the S-layer (γ) (**B**). Long amorphous structures were observed in the vicinity of bacterial, suggesting a desquamation of the S-layer (**C, D**).

**Figure 6:**
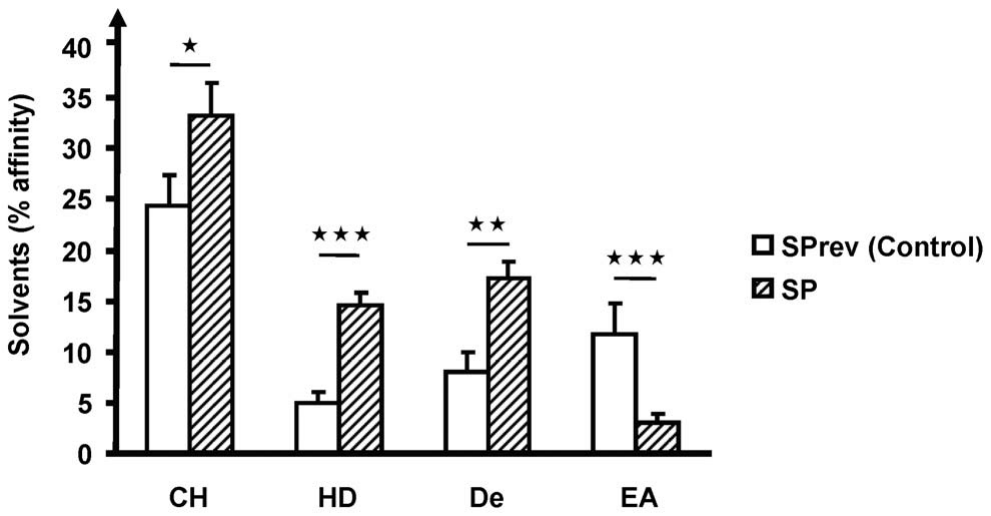
Effect of Substance P reverse (SPrev) and Substance P (10^−6^ M) on the affinity of *Bacillus cereus* to solvents of different polarities. chloroform (CH), hexadecane (HD), decane (De) and ethyl acetate (EA) (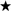 = *P<0.05*, 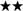 = *P<0.01*, 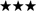 = *P<0.001*). Each value represents the mean ± SEM of three independent experiments.

Bacteria were then examined by TEM. Control *B. cereus* showed a continuous pseudo crystalline surface (Fig. 5A). In contrast, the S-layer of bacteria treated with SP (10^−6^ M) for 13 h appeared interrupted and separated from the membrane by a gap (Fig. 5B). Long amorphous membrane-like structures were observed in the vicinity of the bacterial surface suggesting an important desquamation of the S-layer (Fig. 5C,D). These modifications were associated with a shift in the surface polarity of *B cereus*. Considering that the threshold for the affinity to hexadecane of polar (hydrophilic) bacteria determined by the MATS technique is 20% [25], in our experimental conditions control *B. cereus* behaved as a highly polar micro-organism (5±2% and 8±2% affinity to hexadecane and decane, respectively) (Fig. 6). SP treated bacteria showed a significant increase in affinity to hexadecane (+10±2%) and to decane (+9±1%). In agreement with these observations indicating an increase of surface hydrophobicity of SP-treated *B. cereus*, the affinity of the bacteria to a polar solvent such as ethyl acetate was reduced. Moreover, a comparison of the affinity to the different solvent couples revealed that SP induced a shift in Lewis acid/base character of the bacterial surface. Indeed, in control bacteria the difference in the percentage of affinity between hexadecane and chloroform was about 20% while it decreased to 5% in the couple ethyl acetate/decane. In SP-treated *B. cereus*, these values were 19 and 14% respectively, showing that the bacterial surface evolved from a highly polar and strict Lewis basic character, to a mid polar and Lewis acido-basic character.

### Substance P Increases the Biofilm Formation Activity of *Bacillus Cereus*

**Figure 7:**
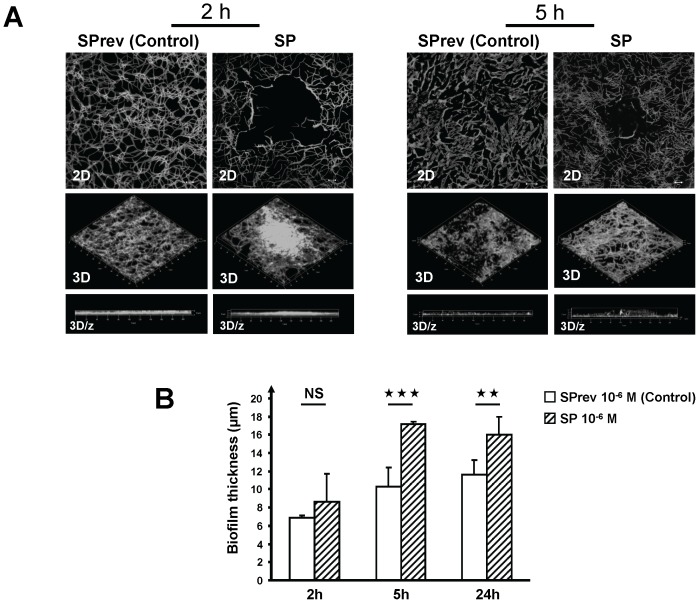
Effect of Substance P reverse (SPrev) and Substance P (SP) (10^−6^ M) on the biofilm formation activity of *Bacillus cereus*. The biofilm formation was observed after 2, 5 and 24 h. (A) Two dimensions (2D) and reconstructed three-dimensions (3D) and ortho cuts (3D/z) images showed that the density of the biofilm was essentially unchanged. (B) In contrast, the thickness of the biofilm was significantly increased after 5 and 24 h inclubation with SP (NS = *non significant*; 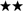 = *p<0.01*; 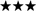 = *p<0.001*).

Surface properties of *Bacillus* have a great influence on their biofilm formation activity [31]. The effect of SP on *B. cereus* biofilm formation was studied by confocal microscopy using GFP-transformed bacteria. First, we verified that the growth kinetic of GFP-pTeTON plasmid transformed bacteria was not modified in the absence or presence of SP or SPrev. After a 2 h treatment with SP (10^−6^ M), the mean surface coverage of the bacteria was unchanged except in specific points where the bacterial mat tended to form blisters (Fig. 7A). The density of the biofilm was also unchanged after 5 h exposition to SP. Yet its thickness was increased on reconstructed 3D and transversal (3D/z) images. As calculated using Zen® software, after 5 h exposition to SP, the thickness of the biofilm rose from a mean value of 10.5 µm to 17.1 µm with SP treated bacteria (Fig. 7B). This effect was maintained, but to a lower degree, in 24 h old biofilms.

### Substance P is Binding on the *Bacillus Cereus* Thermo Unstable Ribosomal Elongation Factor Ef-Tu

**Figure 8:**
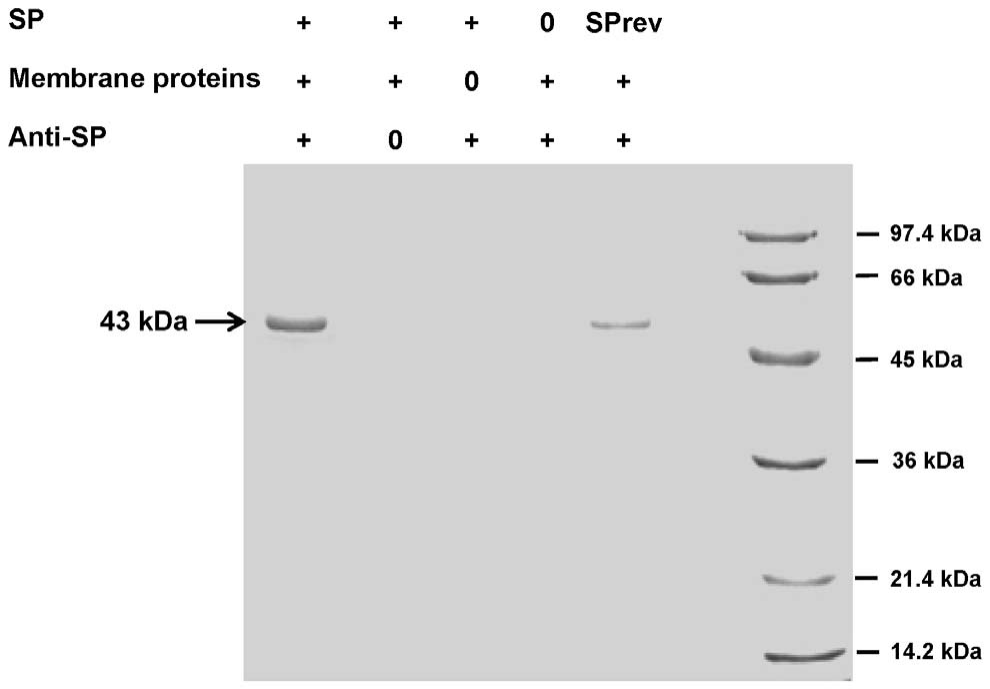
SDS page analysis of *Bacillus cereus* Substance P (SP) binding proteins separated by immunoprecipitation using SP antibody-associated beads. SP was found able to bind on a 43-TOF/TOF as the Thermo Unstable ribosomal Elongation factor Ef-Tu (arrow: 43 kDa). SPrev was also capable of binding to this protein but with a reduced effectiveness. Results are representative of three independent experiments.

We investigated the presence of a SP binding site in the membrane of *B. cereus* considering the properties of SP (ionic charge and peptidic structure) and the very short delay required for the effect of SP on bacteria (<5 min) which suggested an interaction of SP with a surface sensor. A single band, corresponding to a 43 kDa protein, was found as the possible membrane ligand of SP in *B. cereus* (Fig. 8). No trace of non-specific or secondary binding protein was observed. We only noted a partial binding of SPrev on the same 43 kDa protein suggesting that SPrev can bind to the same site with a lower affinity. Nevertheless, SPrev is an inverted peptide and not a scramble, and some epitopes can be preserved. The SP binding site was identified by MALDI-TOF with a MASCOT score of 98 and a coverage percentage of 59% (15 matched fragments) as the Thermo unstable ribosomal Elongation factor (Ef-Tu) (NCBI number NP_830009, 42912 Da, pI = 4.93).

### Substance P also acts on *Staphylococcus Aureus* and *Staphylococcus Epidermidis* Virulence but its Effect can be Antagonized

**Figure 9:**
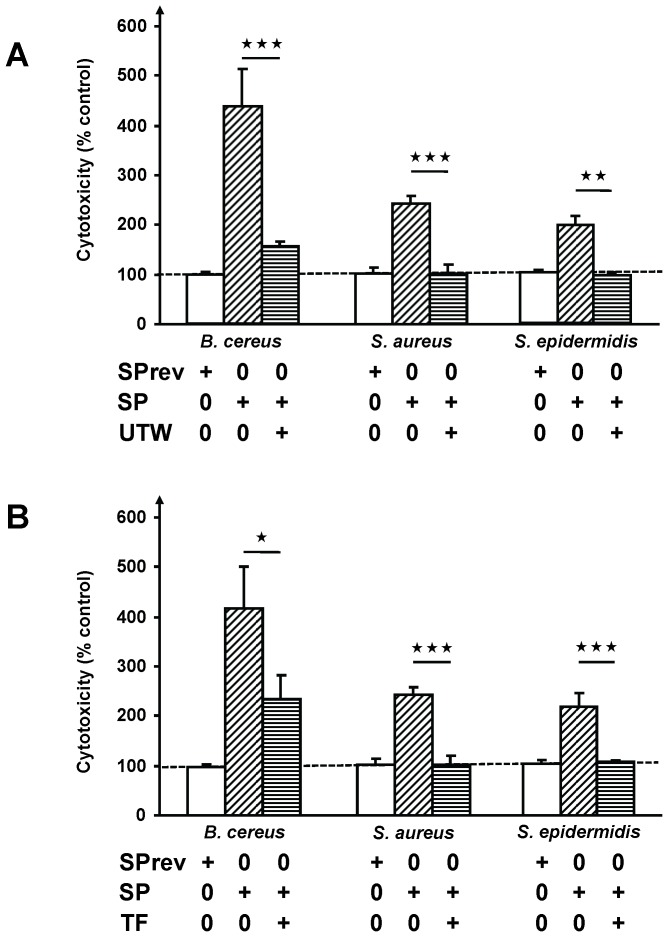
Inhibition of the effects of Substance P (SP) on the cytotoxic activity of *Bacillus cereus*, *Staphylococcus aureus* and *Staphylococcus epidermidis*. (A) Before infection, bacteria were exposed to SP in the presence of thermal water from Uriage-les-Bains (UTW) or (B) Teflose® (TF) (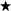 = *p<0.05*, 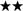 = *p<0.01*, 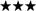 = *p<0.001*).

*B. cereus* is responsible for severe cutaneous infections [32] but *Staphylocci* are considered as the principal Gram positive skin bacteria [2]. The effect of SP on the virulence of *B. cereus* was then compared to that of the peptide on human skin strains of *S. aureus* and *S. epidermidis*. As *B. cereus*, *S. aureus* and *S. epidermidis* responded to a 1 h exposure to SP (10^−6^ M) by a significant increase in their cytotoxicity on HaCaT cells (+242.1±17.2% and +198.5±17%, respectively) (Fig. 9) suggesting that SP should have similar effects over different species of Gram positive bacteria. We also observed that a polysaccharide rich in rhamnose and obtained by fermentation, Teflose® (TF) or a thermal water from Uriage-les-Bains (UTW) were able to antagonize the effect of SP on the three Gram positive bacteria (281.6 and 181% reduction in the activity of SP on *B. cereus* for UTW and TF respectively, and total inhibition in the case of *S. aureus* and *S. epidermidis*).

## Discussion

The skin microbiote is divided in two groups, the resident and the transient microflora. Bacteria of the normal resident microflora establish a quasi-symbiotic relation with the host [33]. In fact, the variations of skin bacterial population or virulence essentially concern the transient microflora that includes mostly opportunistic pathogens. *Bacillus cereus* is a member of this sub-population. In addition to food-born diseases, this bacterium is now recognized as responsible for primary cutaneous infections [32]. This species expresses a large arsenal of virulence factors, including hemolysins, phospholisases, an emetic toxin and pore forming enterotoxins [34] and was selected because of its high infectious potential that made easier the observation of cytotoxicity differences.

The stimulation of *B. cereus* cytotoxicity by SP was high (5.5 fold for SP 10^−6^ M) and rapid (5 min). The controls allowed us to exclude an artefact due to a direct action of SP on keratinocytes. As the reversed sequence peptide SPrev, which has the same amino acid composition as SP was without effect, we can also exclude a metabolic action of the peptide. The assay of cereulide production showed that *B. cereus* MFP01 synthesizes this toxin and then containes the *ces* gene, encoded by a 270 kb plasmide, related to the PXO1 virulence plasmid of *B. anthracis* [35], [36] but the expression of this plasmid is apparently not regulated by SP. *B. cereus* reacted to SP by an over production of the collagenase ColT. This should explain the disorganization of the cell monolayer and is consistent with *in-vivo* studies showing that the role of collagenase in *B. cereus* virulence is essential [37]. The increase of caspase 1 production by keratinocytes exposed to SP-treated bacteria and the morphological changes of the cells are in agreement with an induction of cell necrosis and pyroptosis [29]. However, a massive cell death, as observed with SP treated bacteria, is associated with the release of high amounts of potentially toxic oxidizing compounds. The production of SOD by SP treated *B. cereus* should be a defence reaction against this threat. This response of *B. cereus* to SP was not observed when the bacteria were exposed to SP over a long (13 h) period. In this case, we only noted an increase of the release of S-layer proteins. This result was correlated to the observation of a massive peeling-off of the S-layer of *B. cereus* and to a drop in surface polarity. These events can be interpreted as a general defence reaction of the bacterium against SP. Indeed, although SP did not affect the growth of *B. cereus*, this molecule has structural and functional homologies with antimicrobial peptides and, as recently shown, the bacterial S-layer should act as a barrier against cationic antimicrobial peptides [38]. However, in the present study the concentration of SP was 3 logs below that of the more active antimicrobial peptide (LL-37) [38] and the bacterium reacted not only by releasing S-layer monomers, but by an almost complete desquamation of the S-layer. The *B. cereus* S-layer ultra structure has not been investigated in detail. Assuming that as in other species, in one *B. cereus* cell the S-layer contains an average of 40,000 monomers [39], we calculated that at a concentration of 10^−8^ M (over the threshold of SP effect) less that 1 SP molecule was available to interact with 10 S-layer proteins. Then, SP acts probably indirectly on the stabilization of the polymeric form of the S-layer. The loss of the S-layer induced by SP in *B. cereus* was associated with a decrease in the global surface polarity of the bacterium. As described in *Bacillus anthracis,* cell surface polarity plays a leading role in the adherence to mammalian cells [40] but, to the contrary of our observations, the cytotoxicity of hydrophobic strains is generally lower [41]. The correlation between surface hydrophobicity and virulence in *B cereus* is highly strain dependant [42] but it is also known that the surface of *B. cereus* in biofilms is more polar and hydrophilic than the surface of the planktonic bacteria [43]. However, emetic strains such as *B. cereus* MFP01 only produce a transient biofilm which tends to disorganize after 24 h. As we observed, since the bacterium remained globally hydrophilic, the surface variations are limited [31].

The kinetic of SP on *B. cereus* requires a rapid detection of the peptide; therefore we hypothesized the presence of a surface binding site. Indeed, although untreated *B. cereus* MFP01 showed an intact S-layer protection wall, the S-layer presents multiple pores allowing the secretion of proteins [44]. Additionally, the size of these pores is sufficient to enable the passage of small exogenous peptides similar to SP [38]. We identified a 43 kDa protein, the Thermo unstable ribosomal Elongation factor Ef-Tu, as a specific SP binding site in *B. cereus*. Ef-Tu is present in large excess in bacteria [45]. In addition to its original function at the ribosomal level, it is known that in case of bacterial stress this protein is translocated to the bacterial surface [46] and in *B. anthracis* Ef-Tu was identified as a plasminogen receptor [47]. As there is no sequence homology between SP and plasminogen, Ef-Tu appears as a multifunctional sensor of host signals in *Bacilli*. Because neither SP nor plasminogen are deemed capable of crossing the membrane, Ef-Tu should be associated with a transduction system to trigger the bacterial response. In this regard, it is interesting to note that, in the membrane of *B. subtilis* Ef-Tu is colocalized and should interact with the actin-like protein MreB [48]. The bacterial cytoskeleton does not only show structural homology with its eukaryotic counterpart, but has also similar functions [49], and MreB should play a same role as actin in SP signal transduction in bacteria.

The capacity of detecting SP is shared by other Gram positive bacteria since we observed that *S. aureus* and *S. epidermidis* also reacted to SP by a marked increase in cytotoxicity. Furthermore, the specificity of SP action on *Staphylococci* was the same as in *B. cereus* with an absence of response to SPrev. *Bacilli* and *Staphylococci* are not closely related species, thus we can expect that other Gram positive bacteria should also react to SP. The bacterial diversity in neuropathic diabetic foot ulcer, which is essential in the evolution of the disease, appears dependent of host factors [50]. The decrease of SP expression observed in diabetic foot ulcer [51] should contribute to limit bacterial virulence and consequently host immune reaction, leading to the typically observed low-level chronic infection state. Alternatively, whereas the mean basal concentration of SP in sweat is picomolar [52], local concentrations at the vicinity of the producing cells or nerve terminals should be much higher. The skin concentration of SP is increased in case of atopic dermatitis [53]. Moreover, a 50 fold increase of SP is observed in sweat during depressive disorders [52] and in the case of skin breaches, as measured after surgery, the level of SP in skin exudates can be 8 to 10 nanomolar [54]. The present results should be confirmed using primary adult cells as HaCaT cells are immortalized human keratinocytes, but the threshold of SP effect on *B. cereus* is consistent with the physiological concentrations of SP suggesting that this peptide should actually act in skin as a regulator of the virulence of gram-positive bacteria.

Thermal waters have been known since antiquity to reduce or cure chronic skin inflammation. This is the case of the thermal water from Uriage-les-Bains (UTW). As it is isotonic, this water has no effect on the viability of keratinocytes but when the bacteria were exposed to SP in the presence of 30% UTW, the effect of SP on the virulence of the three bacterial species was reduced or even totally abolished. The mechanism involved was not studied but this water contains a high concentration of anions, particularly chloride (3500 mg/L) and sulfate (2862 mg/L). Then, since SP is a cationic peptide we cannot exclude that it was chelated by these ions and then unable to act on the bacteria. Another compound, Teflose® (TF), a polysaccharide containing rhamnose, glucose and glucuronic acid (BioEurope, Solabia Group) showed similar antagonist effects on SP when it was administered with the peptide. This compound claims to have an anti-adhesive effect on bacteria but because of its charge, it could also act by trapping and sequestering the peptide.

In summary, we showed that SP was able to increase the virulence of bacteria that are commonly present on the skin. The role of SP on skin infections is still controversial. Our study evidenced that the effects of this neuropeptide are not only due to SP effects on inflammation or immunity but could also relate to direct effects on bacteria, by enhancing their virulence. Moreover, the role of bacteria in the pathogenesis of psoriasis, acne or atopic dermatitis is known [33] and our results suggest that the exacerbation of these diseases by SP is not only due to a modulation of inflammation and immunity or to a direct effect of SP on keratinocytes and sebocytes: SP could also exacerbate these diseases by enhancing the virulence of some bacteria then breaking skin defences.
